# Efficacy of mobile health-technology integrated care based on the ‘Atrial fibrillation Better Care’ (ABC) pathway in relation to sex: a report from the mAFA-II randomized clinical trial

**DOI:** 10.1007/s11739-022-03188-2

**Published:** 2023-01-11

**Authors:** Yutao Guo, Bernadette Corica, Giulio Francesco Romiti, Marco Proietti, Hui Zhang, Gregory Y. H. Lip, the mAFApp II trial investigators

**Affiliations:** 1 Department of Pulmonary Vessel and Thrombotic Disease, Medical School of Chinese PLA, Chinese PLA General Hospital, Beijing, China; 2 Liverpool Centre for Cardiovascular Science at University of Liverpool, Liverpool John Moores University and Liverpool Heart and Chest Hospital, Liverpool, UK; 3 Department of Translational and Precision Medicine, Sapienza – University of Rome, Rome, Italy; 4 Department of Clinical Sciences and Community Health, University of Milan, Milan, Italy; 5 Geriatric Unit, IRCCS Istituti Clinici Scientifici Maugeri, Milan, Italy; 6 Department of Clinical Medicine, Aalborg University, Aalborg, Denmark

**Keywords:** Atrial fibrillation, Integrated care, Women, Sex, Outcomes

## Abstract

The Mobile Health Technology for Improved Screening and Optimized Integrated Care in AF (mAFA-II) cluster-randomized trial showed that a mobile health (mHealth)-implemented ‘Atrial fibrillation Better Care’ (ABC) pathway approach reduced the risk of adverse events in atrial fibrillation (AF) patients. Whether this benefit can be applied to both males and females is unclear, especially given the suboptimal management and poorer cardiovascular outcomes in females with AF. In this post-hoc analysis, we performed a sex-stratified analysis of the mAFA-II trial. Between June 2018 and August 2019, adult AF patients were enrolled across 40 centers in China. The primary outcome was the composite of stroke, thromboembolism, all-cause death, and re-hospitalization. The effect of mAFA intervention according to sex was evaluated through adjusted Cox-regression models. Among the 3,324 patients enrolled in the trial, 2,062 (62.0%) patients were males (mean age: 67.5 ± 14.3 years; 1,021 allocated to mAFA intervention) and 1,262 (38.0%) were females (mean age: 70.2 ± 13.0; 625 allocated to mAFA intervention). A significant risk reduction of the primary composite outcome in patients allocated to mAFA intervention was observed in both males (adjusted hazard ratio [aHR] and 95% confidence interval [CI] 0.30 [0.17–0.52]) and females (aHR [95%CI] 0.50 [0.27–0.92]), without statistically significant interaction (*p* = 0.225). Sex-based interactions were observed for other secondary outcomes, including all-cause death (*p* = 0.026) and bleeding events (*p* = 0.032). A mHealth-technology implemented ABC pathway was similarly effective in reducing the risk of adverse clinical events both in male and female patients. Secondary outcomes showed greater benefits of mAFA intervention in men.

**Supplementary Information:**

The online version contains supplementary material available at 10.1007/s11739-022-03188-2.

## Introduction

Atrial fibrillation (AF) is the most common atrial arrhythmia worldwide and is projected to affect 14 million patients in 2060 in Europe alone [1, 2]. Several characteristics influence the epidemiology and natural history of AF; among these, sex is one of the most investigated, with men showing a higher prevalence of AF [3], and women experiencing worse AF-related prognosis [4–6]. The higher thromboembolic risk of women is also reflected by the inclusion of female sex as a risk modifier in the CHA_2_DS_2_-VASc score, used to stratify thromboembolic risk in AF patients [4, 7, 8]

Female AF patients also show a higher burden of AF-related symptoms and a lower quality of life compared to males [9, 10]. Several reasons can explain this, including the lower adoption of rhythm control strategies in females [7], which influences the burden of symptoms reported.

The ‘Atrial Fibrillation Better Care’ (ABC) pathway has been proposed to streamline the implementation of a holistic and integrated care program and improve the prognosis of AF patients, based on three pillars [11]: A, anticoagulation/avoiding stroke; B, better symptom control, and C, optimization of cardiovascular comorbidities, including lifestyle changes. The ABC pathway has been already associated with the reduction of major outcomes in AF patients [12], and is currently recommended as a patient-centered approach by international guidelines [13, 14].

The Mobile Health Technology for Improved Screening and Optimized Integrated Care in AF (mAFA-II) trial evaluated the efficacy of a mobile health (mHealth)-implemented ABC pathway approach (mAFA intervention) in reducing the risk of adverse outcomes among AF patients [15, 16]. The primary analysis showed that mAFA intervention, compared to usual care, reduced the risk of the composite outcomes of ischemic stroke (IS) and systemic thromboembolism (TE), all-cause death, and re-hospitalization [15].

Nevertheless, it is unclear if the effect of mAFA intervention is consistent in both sexes; moreover, the importance of reporting sex-disaggregated data and sex-specific analyses in medical research has been repeatedly underlined, to improve our understanding of sex-based differences and ultimately lead to tailored and effective strategies in both females and males [17, 18]. In this post hoc ancillary analysis, we sought to evaluate the effectiveness of the mAFA intervention in male and female patients with AF, through a sex-stratified analysis of the mAFA-II trial.

## Methods

A detailed description of the rationale, design, and primary results of the mAFA-II trial has been previously published and can be found elsewhere [15, 16]. In brief, the mAFA-II trial was a prospective, cluster-randomized multi-center trial that enrolled adults with AF (≥ 18 years old) across 40 centers in China, that were randomized in a 1:1 ratio to the mAFA intervention or usual care. Patients with mechanical prosthetic valves, those with moderate-to-severe mitral stenosis, and those unable to complete 1 year of follow-up for any reason were excluded. Between June 2018 and August 2019, 1,646 subjects with AF were allocated to mAFA intervention, while 1,678 AF patients were allocated to usual care. The study was approved by the Central Medical Ethic Committee of Chinese PLA General Hospital and by local institutional review boards, and was conducted in accordance with the Declaration of Helsinki and the Consolidated Standards of Reporting Trials (CONSORT) reporting guideline; all the patients gave their written informed consent.

In this post hoc ancillary analysis, we evaluated the effect of mAFA intervention according to the sex of the participants.

### mAFA intervention

The mAFA intervention group implemented the ABC pathway according to the following criteria:

- ‘A’ criterion: anticoagulation prescription according to regular assessment of thromboembolic and bleeding risk, with dose adjustment based on renal and liver function reassessment;
- ‘B’ criterion: regular monitoring of patient-reported symptoms (which were assessed according to the European Heart Rhythm Association classification), and management of symptoms that included antiarrhythmics and rhythm control treatments;
- ‘C’ criterion: active management and treatment optimization of concurrent comorbidities (e.g., hypertension management according to blood pressure monitoring, statin treatment in patients with vascular diseases, etc.). Patients were also provided with educational material and lifestyle recommendations.

Conversely, patients allocated to “usual care” were managed according to local practices.

### Outcomes and follow-up

Follow-up was performed 6 and 12 months after the inclusion. Consistent with the primary trial analysis, the *primary endpoint* was the composite outcome of IS or systemic TE, all-cause death, and re-hospitalization. Other endpoints investigated were TE (defining as IS or other systemic TE), bleeding events (intracranial and/or extracranial), cardiovascular outcomes (recurrent AF, heart failure (HF), acute coronary syndrome), all-cause death, and re-hospitalization.

### Statistical analysis

Baseline characteristics were reported as mean and standard deviation (SD) for normally distributed continuous variables, or median and interquartile range [IQR] for non-normally distributed continuous variables. Binary and categorical variables were reported as frequency and percentage.

For the purpose of this analysis, we analyzed the interactions between sex and the effect of mAFA intervention on the primary and secondary outcomes, using Cox proportional hazard models. All the models were adjusted for age, type of AF, comorbidities (hypertension, diabetes mellitus, CAD, history of HF, history of IS, peripheral artery disease (PAD)), previous AF treatments, and cluster effect. Survival curves were also reported for the risk of the primary composite outcome according to mAFA allocation and sex.

A two-sided *p* value < 0.05 was considered statistically significant. All statistical analyses were conducted using *R* 4.2.0 (R Foundation for Statistical Computing 2020, Vienna, Austria).

## Results

**Fig. 1:**
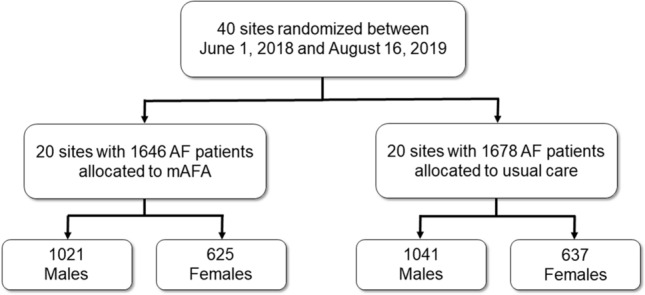
Flowchart of the mAFA-II trial. *AF* atrial fibrillation

Overall, 3,324 patients were enrolled in the mAFA-II trial (Fig. 1); of these, 2,062 (62.0%) were males (mean age ± SD: 67.5 ± 14.3), while 1,262 (38.0%) were females (mean age ± SD: 70.2 ± 13.0). Among males, 1,021 (49.5%) were allocated to mAFA intervention, while 625 females (49.5%) were allocated to mAFA intervention.

Baseline characteristics according to mAFA allocation and sex are reported in Table 1. Compared to the usual care group, male patients in the mAFA intervention were younger and had a lower burden of comorbidities (prior IS, coronary artery disease (CAD), PAD, and prior brain bleeding); among females, those allocated to the mAFA intervention showed a higher prevalence of several comorbidities (HF, prior IS, PAD, and pulmonary hypertension) compared to the control group. Patients in the mAFA intervention were more likely to have received pharmacological cardioversion in both sexes compared to usual care. Treatments prescribed at the time of the enrollment are reported according to sex and allocation in Supplementary Materials, Table S1. In both sexes, patients allocated to mAFA intervention were more frequently treated with non-vitamin K antagonist oral anticoagulants (NOACs) (*p* < 0.001) and less likely to receive antiplatelet drugs.

**Table 1:** Demographic characteristics

	Males			Females		
Variables, *n* (%)	mAFA (*n* = 1021)	Usual care (*n* = 1041)	*p*	mAFA (*n* = 625)	Usual care (*n* = 637)	*p*
Age, mean ± SD	65.2 ± 15.1	69.7 ± 13.2	** < 0.001**	69.7 ± 13.4	70.8 ± 12.5	0.119
Current smoking	140 (13.7)	143 (13.7)	1.000	19 (3.0)	25 (3.9)	0.482
Hypertension	530 (51.9)	576 (55.3)	0.130	378 (60.5)	386 (60.6)	1.000
CAD	373 (36.5)	447 (42.9)	**0.003**	262 (41.9)	277 (43.5)	0.614
Diabetes mellitus	220 (21.5)	231 (22.2)	0.764	161 (25.8)	135 (21.2)	0.065
HF at baseline	198 (19.4)	231 (22.2)	0.131	162 (25.9)	123 (19.3)	**0.006**
Prior ischemic stroke	90 (8.8)	182 (17.5)	** < 0.001**	101 (16.2)	50 (7.8)	** < 0.001**
PAD	89 (8.7)	121 (11.6)	**0.035**	83 (13.3)	51 (8.0)	**0.003**
Renal dysfunction	93 (9.1)	121 (11.6)	0.072	45 (7.2)	51 (8.0)	0.664
Pulmonary hypertension	32 (3.1)	50 (4.8)	0.068	55 (8.8)	33 (5.2)	**0.016**
Liver dysfunction	36 (3.5)	34 (3.3)	0.838	19 (3.0)	14 (2.2)	0.447
Prior thromboembolism	36 (3.5)	43 (4.1)	0.548	18 (2.9)	16 (2.5)	0.818
Prior brain bleeding	14 (1.4)	31 (3.0)	**0.019**	10 (1.6)	7 (1.1)	0.598
Prior other bleeding	29 (2.8)	44 (4.2)	0.113	25 (4.0)	23 (3.6)	0.830
Dilated cardiomyopathy	31 (3.0)	50 (4.8)	0.051	13 (2.1)	11 (1.7)	0.800
Hyperthyroidism	23 (2.3)	28 (2.7)	0.619	14 (2.2)	23 (3.6)	0.202
Hypertrophic cardiomyopathy	17 (1.7)	23 (2.2)	0.461	8 (1.3)	6 (0.9)	0.761
Type of AF			** < 0.001**			** < 0.001**
Unknown	175 (17.2)	64 (6.2)		106 (17.2)	49 (7.7)	
New-onset AF	119 (11.7)	150 (14.4)		76 (12.3)	82 (12.9)	
Paroxysmal AF	419 (41.2)	403 (38.8)		254 (41.2)	257 (40.3)	
Persistent AF	247 (24.3)	290 (27.9)		133 (21.6)	158 (24.8)	
Long-standing AF	33 (3.2)	61 (5.9)		23 (3.7)	40 (6.3)	
Permanent AF	23 (2.3)	72 (6.9)		25 (4.1)	51 (8.0)	
Prior AF treatment						
Pharmacological cardioversion	127 (12.4)	99 (9.5)	**0.040**	86 (13.8)	56 (8.8)	**0.007**
Electrical cardioversion	16 (1.6)	28 (2.7)	0.107	14 (2.2)	7 (1.1)	0.172
AF ablation	114 (11.2)	107 (10.3)	0.562	69 (11.0)	66 (10.4)	0.765
Pacemaker	47 (4.6)	59 (5.7)	0.320	29 (4.6)	26 (4.1)	0.728
LAAO	13 (1.3)	25 (2.4)	0.082	20 (3.2)	5 (0.8)	**0.004**
Scores						
CHA_2_DS_2_-VASc, median [IQR]	2 [1–3]	2 [1–3]	**0.045**	3 [3–4]	3 [2–4]	** < 0.001**
HAS-BLED, median [IQR]	1 [0–2]	1 [1–2]	** < 0.001**	2 [1–2]	1 [1, 2]	0.122

*CAD* coronary artery disease, *HF* heart failure, *IQR* interquartile range, *LAAO* left atrial appendage occlusion, *PAD* peripheral artery disease, *SD* standard deviation; *p* values <0.05 are reported in bold.

### Risk of major outcomes according to mAFA intervention

**Fig. 2:**
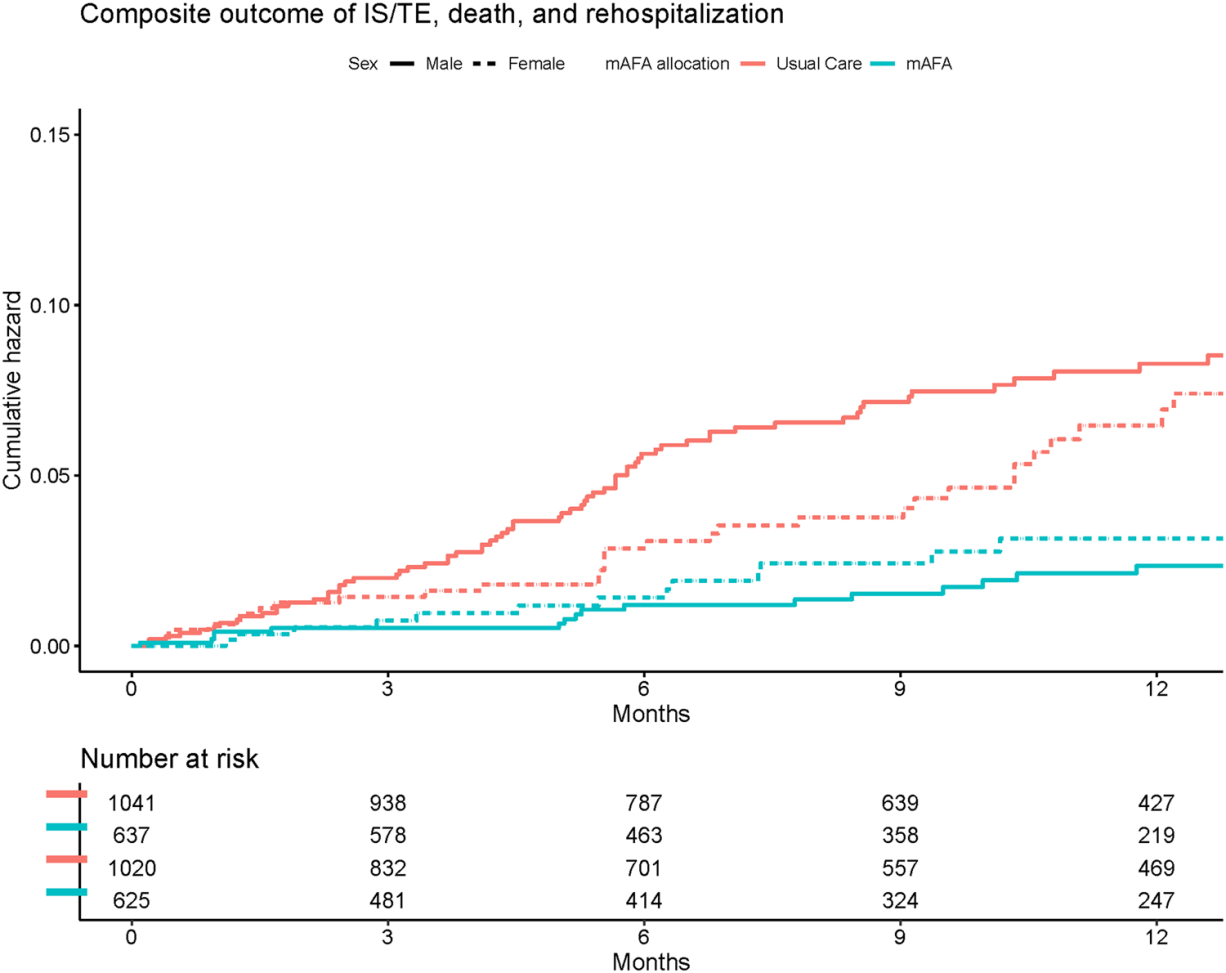
Survival curves for the primary composite outcome, stratified by sex and mAFA allocation. *p* < 0.001 for males, *p* = 0.074 for females. mAFA intervention group = blue, control group = red, male sex = continuous line, female sex = dashed line. *AF* atrial fibrillation, *IS* ischemic stroke, *TE* thromboembolism

**Fig. 3:**
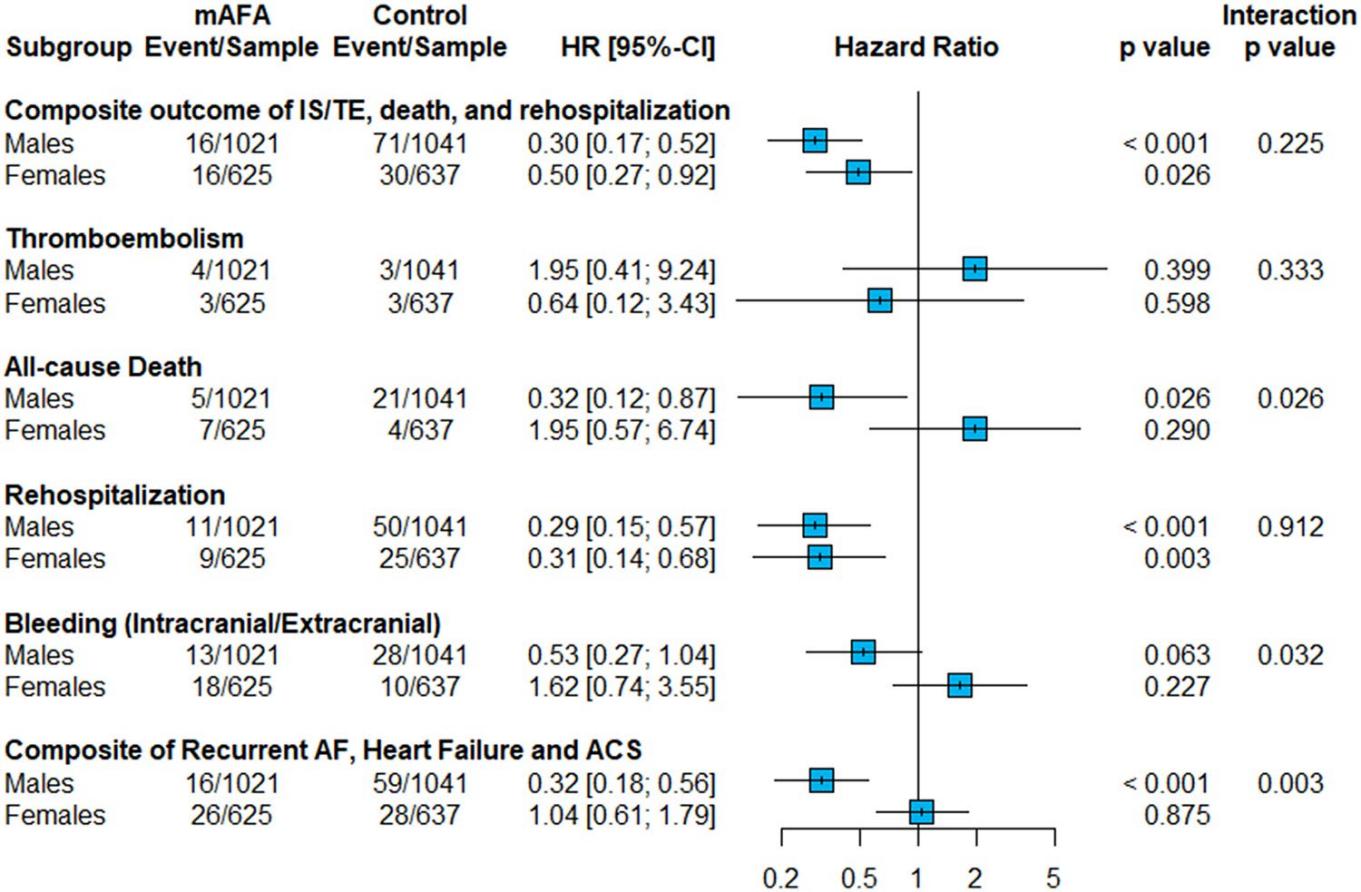
Cox-regression analysis on the interaction between sex and mAFA intervention on the risk of primary and secondary outcomes. *AF* atrial fibrillation; *HR *hazard ratio, *IS* ischemic stroke, *TE* thromboembolism; *adjusted for age, type of AF, previous AF treatments, hypertension, diabetes mellitus, coronary artery disease, prior ischemic stroke, peripheral artery disease, chronic heart failure, and cluster factor

Survival curves for the primary composite outcome according to sex and mAFA allocation are reported in Fig. 2, while the results of the analysis on the interaction between sex and the effect of mAFA intervention on the risk of primary and secondary outcomes are reported in Fig. 3. Both male and female patients allocated to mAFA intervention showed a reduced risk of the *primary composite outcome* of IS/TE, all-cause death and re-hospitalization (aHR [95%CI] 0.30 [0.17–0.52] and 0.50 [0.27–0.92] for males and females, respectively), with no significant sex-based interaction (p_int_ = 0.225). Among the exploratory secondary outcomes, a statistically significant sex-based interaction was observed for the all-cause death (p_int_ = 0.026), bleeding events (p_int_ = 0.032) and the composite outcome of recurrent AF, heart failure, and ACS (p_int_ = 0.003); for all these outcomes, the effect of mAFA intervention in reducing the risk of the events was higher in male patients. No statistically significant interaction was observed for the risk of thromboembolism and re-hospitalizations alone.

## Discussion

In this ancillary analysis of the mAFA-II trial, our principal findings are as follows: (i) the mAFA intervention reduced the risk of the primary composite outcome of IS, TE, all-cause death, and re-hospitalization in both sexes; (ii) the magnitude of the risk reduction appeared higher among males; and (iii) a sex-based interaction was observed for some of the exploratory secondary outcomes, including all-cause death and bleeding events, with the effect of mAFA intervention being higher among male patients.

There has been growing interest in the potential sex-based differences in AF patients, from the pathophysiology and the accumulation of risk factors to the risk of adverse outcomes, including stroke [6, 19, 20]. Notwithstanding the under-representation of women in clinical trials [21], female AF patients experience a significant burden of symptoms [22, 23] and worse prognosis [6, 24], while also being often undertreated in clinical practice [23, 25]. Therefore, females represent one key group of AF patients with an unmet need for better management and improved outcomes.

Recent international guidelines [13, 14] have underlined the need for an integrated care approach to optimize the management of AF patients, and the ABC pathway has been proposed to streamline such a holistic bundle of care [11]. While the ABC pathway has been already proven effective in improving outcomes among AF patients [12, 26, 38﻿], previous subgroups’ analysis of retrospective studies have suggested that potential sex-based differences may exist in the efficacy of the ABC pathway, with women potentially experiencing a lower magnitude of effect [27].

In this post hoc analysis from the mAFA-II trial, we showed how a mHealth-technology implemented ABC pathway consistently reduces the risk of the primary composite outcome of IS/TE, all-cause death, and re-hospitalizations in both sexes, without a statistically significant sex-based interaction. Nevertheless, we observed a trend toward a higher magnitude of risk reduction among male patients, and this finding was consistent with the analysis of the exploratory secondary outcomes, which showed a sex-based interaction for the effect of the ABC pathway on the risk of all-cause death, bleeding events, and the composite of non-fatal cardiovascular outcomes.

Several hypotheses may contribute to the results observed. First, in our study, male patients allocated to mAFA intervention were younger than those allocated to usual care, and with an overall lower burden of comorbidities at baseline, including CAD, PAD, and history of previous IS and bleeding events. On the other side, women allocated to mAFA showed higher prevalence of HF, PAD. and a history of IS. Taken together, while these imbalances in baseline characteristics can be explained by the cluster randomization design of the trial, they may have contributed to the higher magnitude of the effect observed among male patients compared to females allocated to mAFA intervention.

Moreover, both males and females allocated to mAFA intervention more frequently received OAC—and, specifically, NOACs—when compared to those who received usual care. The higher uptake of OAC can be seen both as a direct effect of the implementation of the mAFA intervention in these patients (with optimisation of stroke prevention as part of the mHealth-implemented ABC pathway), and one key determinants of the beneficial effects of mAFA in these patients. These findings reinforce the hypothesis that a holistic or integrated care approach improves management of AF patients, leading to better outcomes.

Nonetheless, it has already been shown that women may present with atypical symptoms of AF [28], which may contribute to challenges in the diagnosis and management; furthermore, several reports have identified female sex as being associated with less efficacy of rhythm control strategies in AF patients, including catheter ablation, and also higher rate of procedural complications [29–32]. Taken together, these data suggest that achievement of symptoms control may be more challenging in women than in men, and this may lead to worse quality of life and, ultimately, worse prognosis.

Finally, the role of social determinants of health in influencing the natural history and outcomes in AF is increasingly recognized [33, 34], and currently represents one of the most unmet needs in the management of AF patients. The detrimental impact of socioeconomic factors on cardiovascular outcomes, especially among women, has been already established [35]; moreover, women are disproportionately affected by social disparities and social deprivation, thus leading to overall low access to health resources, low quality of life, and worse prognosis [36]. Consistently, a recent study has shown how low educational status, low income, and living alone were all associated with a lower uptake of AF ablation after an incident AF diagnosis [37], further underlying how these issues are crucial—although often overlooked—in ensuring optimal management and prognosis in AF patients. Of note, the ABC pathway is not specifically designed to target the social determinants of health, and this may contribute to the findings observed in our study, especially considering how these issues disproportionately affect women.

Overall, while confirming the efficacy of the ABC pathway in both sexes for the primary composite outcome, our findings are consistent with the previously reported trend of a potential sex-based difference in the efficacy of the ABC pathway [27]. These observations have several important clinical implications: first, the implementation of a mHealth technology-implemented ABC pathway to streamline an integrated care approach may help in reducing the health gap between male and female AF patients, otherwise wider, and therefore the implementation of this approach should be encouraged in both sexes. Notwithstanding, women may experience an attenuated effect of the ABC pathway for several reasons, including differences in the pathophysiology and clinical presentation of AF, and the higher impact of non-traditional risk factors (including social determinants of health), which require tailored strategies to reduce the risk of adverse outcomes in females. Specifically, increasing the awareness of the sex-based differences in the natural history of AF, and implementing strategies to tackle the social inequalities and barriers which may influence the access to care and prognosis of women, may represent two of the most crucial interventions that may reduce the inequalities between sexes. While the ABC pathway represents the cornerstone of treatment for AF patients, the combination of an integrated care approach with sex-tailored strategies may represent the key to further improving outcomes in AF patients, especially in females.

### Strengths and limitations

Our study is the first analysis to provide a sex-stratified analysis on the efficacy of a mHealth-implemented ABC pathway, and will be particularly useful to inform sex-specific recommendations and guidance, especially given the urgent need for sex-disaggregated data in this scenario [21]. Furthermore, our results were largely consistent with the primary analysis of the trial, contributing to the reliability of our estimates.

Nonetheless, our study has some limitations. First, this was a post hoc analysis of a cluster-randomized trial and may lack statistical power for some of the outcomes investigated, and for the specific subgroups examined about which the trial was not originally powered. Second, there were some imbalances on the baseline characteristics in both males and females allocated to mAFA intervention vs. usual care. While this is compatible with the randomized cluster design of the trial, this may have contributed, at least partly, to the results observed. Third, we were unable to evaluate the role of the social determinants of health (SDOH) in determining the results observed. Further studies are required to evaluate the role of SDOH and the potential sex-based differences in this clinical context. Fourth, although we adjusted our analyses for several potential moderators, we cannot exclude the effect of unaccounted confounders on the findings observed.

## Conclusion

In this post hoc analysis of the mAFA-II trial, we found that a mHealth-technology implemented ABC pathway was similarly effective in reducing the risk of adverse clinical events both in male and female patients. Secondary outcomes showed greater benefits of mAFA intervention in men.

## Supplementary Information

Below is the link to the electronic supplementary material.

Supplementary file1 (DOCX 19 KB)

## Data Availability

Data supporting the current study are available from the corresponding author upon reasonable request.
