# Supplementary material for: Efficacy of mobile health-technology integrated care based on the ‘Atrial fibrillation Better Care’ (ABC) pathway in relation to sex: a report from the mAFA-II randomized clinical trial
Source: Intern Emerg Med. 2023 Jan 11;18(2):449–56. doi: 10.1007/s11739-022-03188-2 (PMC10017580; doi:10.1007/s11739-022-03188-2)
Supplement: Supplementary file 1 — Supplementary file1 (DOCX 19 KB) [file 11739_2022_3188_MOESM1_ESM.docx]

**Efficacy of Mobile Health-Technology Integrated Care based on ‘Atrial Fibrillation Better Care’ (ABC) Pathway in relation to Sex: A report from the mAFA-II Randomized Clinical Trial**

**Brief Title:** Sex and mHealth Integrated Care in AF

*Supplementary Materials*

Yutao Guo, MD PhD^1,2^*, Bernadette Corica, MD^2,3*^ Giulio Francesco Romiti, MD^2,3^, Marco Proietti, MD PhD^2,4,5^, Hui Zhang, MD^1^, Gregory Y. H. Lip, MD^1,2,6^;

on behalf of the mAFApp II trial investigators

^1^Department of Pulmonary Vessel and Thrombotic Disease, Medical School of Chinese PLA, Chinese PLA General Hospital, Beijing, China; ^2^Liverpool Centre for Cardiovascular Sciences at University of Liverpool, Liverpool John Moores University and Liverpool Heart & Chest Hospital, Liverpool, United Kingdom; ^3^Department of Translational and Precision Medicine, Sapienza – University of Rome, Rome, Italy; ^4^Department of Clinical Sciences and Community Health, University of Milan, Milan, Italy; ^5^Geriatric Unit, IRCCS Istituti Clinici Scientifici Maugeri, Milan, Italy; ^6^Department of Clinical Medicine, Aalborg University, Aalborg, Denmark.

*joint first authors

Corresponding Author

**Prof. Gregory Y H Lip**

Liverpool Centre for Cardiovascular Science, University of Liverpool

William Henry Duncan Building, 6 West Derby St, Liverpool L7 8TX, United Kingdom

E‐mail: [gregory.lip@liverpool.ac.uk](mailto:gregory.lip@liverpool.ac.uk)

**Supplementary Table S1.** Treatment at baseline according to mAFA allocation and sex.

|  | **Males** | | | **Females** | | |
| --- | --- | --- | --- | --- | --- | --- |
| **Variables, n (%)** | **mAFA (n=1021)** | **Usual Care (n=1041)** | **p** | **mAFA (n=625)** | **Usual Care (n=637)** | **p** |
| **Pathway A** | | | | | | |
| Warfarin | 21 (2.1) | 79 (7.6) | **<0.001** | 34 (5.4) | 65 (10.2) | **0.003** |
| NOACs | 604 (59.2) | 399 (38.3) | **<0.001** | 429 (68.6) | 269 (42.2) | **<0.001** |
| Aspirin | 74 (7.3) | 179 (17.2) | **<0.001** | 55 (8.8) | 98 (15.4) | **<0.001** |
| Clopidogrel | 53 (5.2) | 92 (8.8) | **0.002** | 33 (5.3) | 37 (5.8) | 0.774 |
| Ticagrelor | 3 (0.3) | 19 (1.8) | **0.002** | 6 (1.0) | 7 (1.1) | 1.000 |
| **Pathway B** | | | | | | |
| Beta-Blockers | 263 (25.8) | 189 (18.2) | **<0.001** | 255 (40.8) | 133 (20.9) | **0.005** |
| Propafenone | 17 (1.7) | 25 (2.4) | 0.306 | 13 (2.1) | 22 (3.5) | 0.189 |
| Amiodarone | 126 (12.3) | 51 (4.9) | **<0.001** | 74 (11.8) | 28 (4.4) | **<0.001** |
| **Pathway C** | | | | | | |
| ACEI | 84 (8.2) | 123 (11.8) | **0.008** | 105 (16.8) | 95 (14.9) | 0.401 |
| ARB | 135 (13.2) | 139 (13.4) | 0.982 | 153 (24.5) | 88 (13.8) | **<0.001** |
| Diuretics | 104 (10.2) | 183 (17.6) | **<0.001** | 100 (16.0) | 123 (19.3) | 0.142 |
| Calcium Channel Blockers | 155 (15.2) | 132 (12.7) | 0.115 | 143 (22.9) | 97 (15.2) | **0.001** |
| Statins | 308 (30.2) | 321 (30.8) | 0.778 | 315 (50.4) | 224 (35.2) | <0.001 |
| Digoxin | 48 (4.7) | 60 (5.8) | 0.325 | 45 (7.2) | 27 (4.2) | 0.032 |
| Nitrate | 98 (9.6) | 147 (14.1) | **0.002** | 94 (15.0) | 91 (14.3) | 0.765 |

**Legend:** ACEI= Angiotensin converting enzyme inhibitor; ARB= Angiotensin receptor blockers NOACs= Non-vitamin K antagonist Oral Anticoagulants.
